# Three-Dimensional Printing and Personalized Bioceramic Scaffolds for Dental and Maxillofacial Applications: A Narrative Review

**DOI:** 10.3390/dj14040237

**Published:** 2026-04-15

**Authors:** Seyed Ali Mostafavi Moghaddam, Hamid Mojtahedi, Amirhossein Bahador, Lotfollah Kamali Hakim, Hamid Tebyaniyan

**Affiliations:** 1 School of Dentistry, Tehran University of Medical Sciences, Tehran 1439955991, Iran; 2 Oral and Maxillofacial Surgery Department, Craniomaxillofacial Research Center, Tehran University of Medical Sciences, Tehran 1411713135, Iran; 3 UCLA School of Dentistry, University of California, Los Angeles, CA 90095, USA; 4 Department of Oral and Maxillofacial Surgery, School of Dentistry, AJA University of Medical Sciences, Tehran 1411718541, Iran; 5 Science and Research Department, Islimic Azade University, Tehran 1651153311, Iran

**Keywords:** 3D printing, additive manufacturing, personalized scaffolds, bioceramics, calcium phosphate, calcium silicate, bioactive glass, dental tissue engineering, maxillofacial reconstruction, regenerative dentistry

## Abstract

**Background/Objectives:** Bioceramic scaffolds with complex geometries and customized mechanical and biological properties can now be produced via 3D printing, revolutionizing dental and maxillofacial tissue engineering. This review discusses the recent progress in 3D printing technologies applied to bioceramic scaffolds for dental and maxillofacial reconstruction. **Methods:** A comprehensive literature search was conducted across major electronic databases, including Scopus, PubMed, ScienceDirect, and Web of Science. Peer-reviewed articles published between 2015 and 2026 were considered for inclusion. Several 3D printing methods can be used to create bioceramic or composite scaffolds for the regeneration of dental, oral, or maxillofacial tissues. **Results:** Additive manufacturing enables customization of bioceramic scaffolds. This report emphasizes the osteoconductive properties, biodegradability, and compatibility of calcium phosphate, bioactive glass, and calcium silicate ceramics. **Conclusions:** This review helps to determine how 3D-printed bioceramics can be optimized for dental and maxillofacial applications tailored to specific patients.

## 1. Introduction

A persistent clinical challenge is the treatment of dental and maxillofacial bone defects caused by trauma, tumor resection, congenital malformations, infection, or tooth loss. Due to its osteogenic, osteoinductive, and osteoconductive properties, autologous bone grafting remains the gold standard. Clinical applications are limited by donor-site morbidity, limited graft availability, prolonged operative time, and unpredictable resorption. The search for synthetic alternatives capable of providing structural support while promoting functional bone regeneration has been driven by these shortcomings [1,2].

A number of synthetic candidates for bone regeneration have emerged in recent years, including calcium phosphates, calcium silicates, and bioactive glasses, due to their intrinsic bioactivity, compositional similarity to bone minerals, and ability to degrade at rates that can be controlled [3,4,5]. The traditional methods of fabricating scaffolds, however, have been shown to be less efficient at achieving precise architectural control, adequate pore interconnectivity, and patient-specific geometric conformity, all of which are essential for vascularization [6,7].

Through the use of additive manufacturing (AM), also known as three-dimensional (3D) printing, bone scaffolds can be designed and manufactured with controlled porosity, geometric lattices, and external morphologies. Anatomically customized scaffolds can be produced via AM using cone-beam computed tomography (CBCT) or computed tomography (CT) data, thereby facilitating defect adaptation, optimizing load distribution, and reducing the need for resections during the operation. The 3D-printed constructs support cell attachment, migration, and guided tissue formation more effectively than traditional particulate grafts [8,9].

Although technology has made significant progress, significant challenges remain in material science, biology, and translation. Pure ceramics are intrinsically brittle and have low fracture toughness, which makes them unsuitable for heavy-duty maxillofacial applications. Lattice architecture, pore size distributions, surface topography, ionic composition, and degradation behavior also influence scaffold performance. These factors influence osteogenesis, angiogenesis, immune response, and long-term remodeling. There are several emerging strategies to overcome these biological and mechanical limitations, including composite scaffold fabrication, ion-doped ceramics, mesoporous bioactive glasses, and hybrid ceramic–polymer printing [10,11,12].

The clinical translation process must also address sterilization protocols, reproducibility, workflow validation, regulatory compliance, and economic feasibility. To ensure the safety and efficacy of personalized scaffolds, standardized digital workflows and robust preclinical and clinical validation are required. It complicates comparisons between studies and hinders the adoption of evidence-based clinical practices because there are no uniform evaluation metrics [6,13].

Advancements in 3D-printed bioceramic scaffolds have been reported in numerous studies. Still, there remains limited synthesis that integrates material chemistry, scaffold architecture, biological performance, and translational considerations in dental and maxillofacial reconstruction [3,14,15]. This review discusses the recent progress in 3D printing technologies applied to bioceramic scaffolds for dental and maxillofacial reconstruction.

## 2. Methods

The objective of this study was to provide an updated and comprehensive overview of advances in 3D printing technologies and personalized bioceramic scaffolds for dental and maxillofacial applications. A thorough and unbiased synthesis of the available scientific evidence and recent developments was achieved using the methodology. A comprehensive literature search was conducted across major electronic databases, including Scopus, PubMed, ScienceDirect, and Web of Science. The search covered studies published between 2015 and 2026. The following keywords were used: (“3D printing” OR “additive manufacturing” OR “rapid prototyping”) AND (“bioceramic” OR “calcium silicate” OR “hydroxyapatite” OR “bioactive glass”) AND (“scaffold” OR “bone regeneration” OR “tissue engineering”) AND (“dental” OR “maxillofacial” OR “oral surgery” OR “craniofacial reconstruction”).

Inclusion criteria: To identify additional studies that may have been missed in database searches, these studies were also manually searched in the reference lists of relevant articles and recent review papers. Peer-reviewed articles published between 2015 and 2026 were considered for inclusion. The studies are written in English. Several 3D printing methods can be used to create bioceramic or composite scaffolds for the regeneration of dental, oral, or maxillofacial tissues. In addition to experimental studies, clinical studies were also considered. Furthermore, review articles, meta-analyses, and book chapters on 3D-printed bioceramics were included to provide context and support for discussion.

Exclusion criteria: A non-peer-reviewed publication, an abstract from a conference, or an editorial was excluded. Applications not related to dentistry or craniofacial surgery. Articles that do not involve bioceramics or hybrid scaffolds. Material reports that focus solely on polymeric or metallic materials without any ceramic components.

Independent reviewers screened the abstracts and titles for eligibility. An evaluation of potentially relevant articles was conducted by retrieving and evaluating their full texts. Discussions were held to resolve discrepancies. Detailed information regarding bioceramic materials, 3D printing techniques, scaffold properties, biological performance, and clinical and preclinical application sites was extracted from each included study.

## 3. 3D Printing Technologies for Bioceramic Scaffolds

Bone tissue engineering uses scaffolds to support cell attachment and proliferation. To regenerate bone tissue, scaffolds must mimic its chemical, hierarchical, and mechanical properties. Bioceramic scaffolds are increasingly used for bone regeneration [7,16,17]. Clinically, reconstructing critical-size bone defects remains challenging. There are several drawbacks to autogenous and allogeneic grafts in clinical practice, including limited availability, donor-site complications, and immune rejection, which is why researchers have developed artificial bone substitutes based on various materials and fabrication techniques. Bioceramic-based replacements are biocompatible and can also degrade concurrently with the formation of new bone. Personalized 3D bioceramic scaffolds are also frequently fabricated using 3D printing technologies that can accurately reproduce native bone structures. Through bioprinting, cells can be integrated into scaffolds to produce organoids that mimic organ structure and function simultaneously [18,19].

### 3.1. Extrusion-Based Printing (Robocasting and Direct Ink Writing)

A layer-by-layer fabrication technique, extrusion-based additive manufacturing, commonly known as Direct Ink Writing (DIW) or robocasting, involves forcing a paste-like ink through a nozzle and depositing it along a programmed toolpath to create three-dimensional structures. It is based on the rheological design of the ink, which allows it to flow through the nozzle while retaining its shape immediately after deposition, enabling printing of high-fidelity, self-supporting filaments and highly porous lattice geometries [20,21,22]. Printability and material properties are both determined by the DIW ink formulation. A ceramic or composite ink is usually composed of a high solid loading, a liquid medium, dispersants and binders for tuning interparticle interactions, and rheology modifiers to maintain shape. To reduce defects such as nozzle clogging, filament spreading, and poor interlayer bonding, it is crucial to optimize these parameters [23,24,25]. Besides process variables, hardware choices can also significantly impact outcomes. Extrusion pressure, printing speed, and toolpath strategy influence filament dimensions, porosity, and surface finish. Inks formulated with solvents can be sensitive to temperature and humidity. Ceramic systems that print “green” bodies typically require careful drying, binder burnout (debinding), and sintering after printing to achieve densification and mechanical integrity; these steps introduce shrinkage and can change the pore architecture, so print designs must anticipate the evolution of the post-process. Researchers are working on standardizing parameter optimization and in-process monitoring to improve reproducibility and industrial scalability [20,26].

DIW/robocasting offers low equipment costs, compatibility with a wide range of chemistries, and straightforward multi-material capabilities that enable spatial gradients, embedded phases, and architected porosities. A number of fields have benefited from these characteristics, including tissue engineering scaffolds, porous filters and catalyst supports, energy materials (electrodes), and custom ceramics for structural and biomedical applications. There are still a number of technical limitations: maintaining dryness and preventing shrinkage in particle-rich inks, and ensuring interlayer mechanical performance after sintering remain major challenges [27,28,29]. Multifunctional inks, hybrid printing strategies, and improved rheological characterization methods are recent advancements. In both research and clinical settings, the development of standard processes and data-driven parameter maps is accelerating the translation of DIW from laboratory demonstrations to reproducible manufacturing. In the field of dental and maxillofacial biomaterials, DIW’s ability to produce patient-specific porous scaffolds and complex ceramic shapes with tunable porosity offers promising options for bone graft substitutes and custom implants, provided post-sintering dimensional changes and biocompatibility are carefully managed [27,30,31].

There are, however, limitations to ceramic printing based on extrusion. Because drying and shrinkage can distort geometry, it is challenging to achieve high density after sintering while maintaining design macroporosity. Additionally, high solids loadings require higher ink viscosity and greater extrusion forces for robust sintered parts. To prevent cracking or loss of interconnectivity, post-processing is critical for each ceramic chemistry. To achieve sufficient toughness and clinical handling properties for load-bearing craniofacial applications, printed bioceramics are often hybridized with polymers [32,33]. Extrusion-based methods have been successfully used in dentistry and maxillofacial surgery to produce patient-specific grafts based on CT-derived anatomical and pore-size information. Based on recent research, robocast/DIW scaffolds made of calcium phosphate, bioactive glass, or composite formulations support osteoconduction and can be loaded with bioactive agents to stimulate bone growth. With careful materials and thermal-processing design, they can provide customized craniomaxillofacial reconstruction [6,8,34].

### 3.2. Stereolithography and Digital Light Processing (DLP)

Stereolithography (SLA) and Digital Light Processing (DLP) build layers by layer using a liquid photosensitive resin and a light source. A focused UV laser beam is used in SLA to trace each layer’s cross-section, while a pattern is projected onto the resin surface in DLP. Resin is solidified at a fine optical scale rather than being extruded or deposited as in powder- or filament-based AM methods [35,36,37]. For temporary restorations and models, photopolymer dental resins were initially popular. However, vat photopolymerization has been extended to composite resins, ceramic-filled resins, and indirect bioceramics: ceramic particles can be dispersed in a photopolymer matrix, printed with SLA/DLP, and then post-processed to produce dense ceramic bodies with complex geometries, such as alumina, zirconia, or hydroxyapatite. Among dental applications, DLP’s speed and accuracy make it ideal for producing crowns, surgical guides, splints, and detailed study models [32,38]. It offers many advantages, including very high feature resolution, a fine surface finish, and compatibility with a wide range of resin and particle-filled systems. It is difficult to manufacture truly load-bearing, certified biomaterials in direct photopolymer form due to limited availability, shrinkage and warpage during thermal treatment and post-cure for ceramic parts, and scale constraints. Due to the VAT size and optical projector/laser characteristics, large parts remain challenging. To move from prototyping to clinical use, process parameters must be carefully validated [38,39,40,41]. Among the recent projects are expanding printable materials libraries (bioresins, ceramic slurries, and functional composites), increasing the resolution and uniformity of projectors/lasers, and integrating multimaterial or cell-friendly formulations for tissue engineering and bioprinting. The regulatory acceptance and standardization of dental/medical devices printed by SLA/DLP remain active areas of development as these technologies move beyond lab demonstrations and into routine clinical production [42,43,44].

SLA and DLP are vat-photopolymerization techniques increasingly used to construct clinically specific bioceramic scaffolds. Using these methods, ceramic-filled photopolymer slurries are patterned layer-by-layer (laser-scanning in SLA, whole-layer projection in DLP) and then debound and sintered to create dense or porous ceramic scaffolds (e.g., hydroxyapatite, β-TCP, bioglass, zirconia). Since DLP combines very high resolution (feature sizes down to tens of microns) with fast per-layer curing, it is widely used for dental/maxillofacial scaffolds. This allows accurate reproduction of craniomaxillofacial defects, including complex trabecular geometries and small anatomical features [45,46,47]. Photocurable organic matrixes loaded with ceramic powder are used in ceramic stereolithography. The particle size distribution, dispersant concentration, rheology modifier concentration, and photoinitiator concentration must be carefully controlled to maintain adequate cure depth and layer adhesion after sintering, thereby minimizing shrinkage and achieving acceptable mechanical properties. Zirconia and HA slurries are printable at 50–56 vol% with tailored dispersants and curing models, according to recent optimization studies [48,49].

Each cross-section is traced by a UV laser, yielding extremely fine, continuous features. By projecting pixelated images of each layer onto a micromirror or LCD panel, DLP cures entire layers simultaneously, giving high throughput for parts with wide cross-sections and excellent replication of small, repeatable patient-specific geometries. Ceramic particles scatter light, and pixelation sets practical limits on DLP feature sizes [37,50,51]. With DLP, multimodal porosity is fabricated, including macropores that support vascularization and bone ingrowth, as well as micropores from particle packing and sintering that increase surface area and bioactivity. A variety of studies have reported compressive strengths of HA-filled TCP scaffolds that are suitable for craniofacial bone repair and osteogenesis in vitro [34,52,53]. In 3D bioprinting, bioinks are created by combining viable cells with appropriate biomaterials to build complex, heterogeneous structures that support cell–cell and cell–matrix interactions. By replicating biological tissues, the specific functions can be reproduced in biomedical applications. 3D bioprinting solutions based on SLA/DLP have favorable properties and can fabricate highly complex constructs, making them valuable for tissue modeling and regenerative medicine. Three-dimensional in vitro models have been extensively studied in the biomedical field for their utility in drug screening and in understanding pathogenesis. The development of bioprinting techniques over the past decade has led to the replacement of animal models in preclinical drug development, which are often expensive and uncertain. SLA/DLP-based bioprinting methods are being used to reproduce delicate architectures, complex compositions, and functions of human tissues [54,55,56]. Using SLA/DLP, complex geometries and advanced tissue replacements, ranging from the vasculature to neural networks, can be constructed with high resolution for implantable scaffolds and advanced tissue replacements with tunable physicochemical properties. Biomimetic scaffolds made from 3D-bioprinted materials can support functional reconstruction based on their physicochemical features [54,55,56].

**Figure 1:**
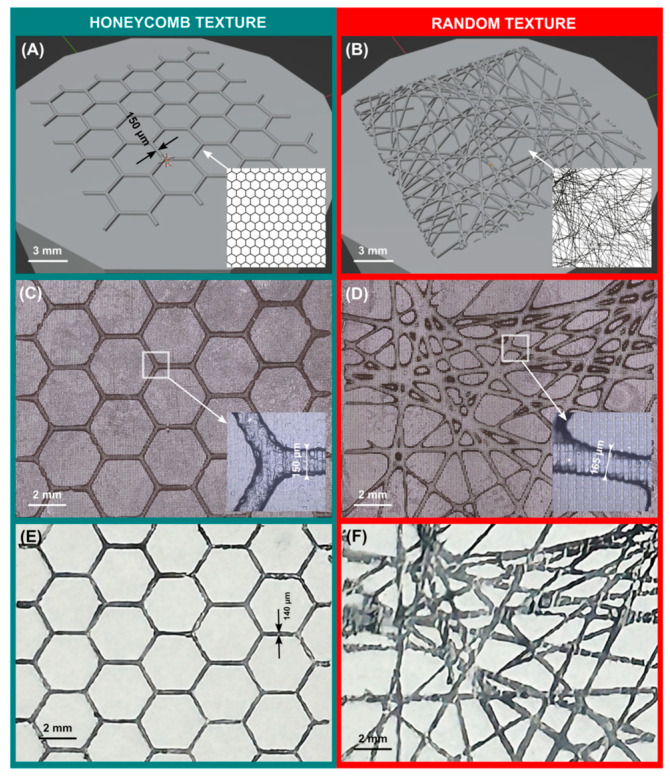
3D mesh model of a stamp with a honeycomb texture (**A**) and a random texture (**B**), with the imported image inset into each image. (**C**) The honeycomb texture and (**D**) the random texture on the 3D-printed stamp surface, with a magnified view of a selected detail in each inset. Ink pattern on paper as seen through an optical microscope when using the stamps with honeycomb texture (**E**) and random texture (**F**). The stamping was performed for 10 s under a load of 450 g [54].

Stereolithographic and digital light-processed three-dimensional (3D) printed provisional resins were evaluated by Wadhwani et al. (2022) [57]. A mean marginal gap was observed on the mesiobuccal surface of the first premolar for the DLP group, while a minimal marginal gap was observed for the SLA group. DLP samples displayed statistically significantly higher mean marginal gaps than SLA samples. There was no significant difference in surface roughness among the samples. After polishing, Rz (roughness depth) showed a statistically significant difference. The marginal adaptation of temporary resin FPDs printed via SLA was significantly better than that of those printed via DLP. There was no significant difference in surface roughness between samples [57]. Bail et al. (2024) used polygonal modeling software and photopolymerized resin to produce a surface-textured part [54]. They developed a universal method to predict the dimensional accuracy of a model file log. Characterization of the printed components was conducted using a confocal imaging microscope. According to the setup, the mesh size had to be reduced to 10% of the smallest feature size, and the textured layer had to be overexposed. A functional stamp with regular (honeycomb) and random textures was successfully produced. By projecting imported images, honeycomb and random images are obtained, as shown in Figure 1. A sufficient overexposure applied to the textured layers resulted in the desired textures on both stamps. Through these insights, innovative patterns and textures will be produced quickly and cost-effectively for hobby, industrial, and biomedical applications [54].

### 3.3. Selective Laser Sintering (SLS) and Selective Laser Melting (SLM)

In powder-bed fusion, SLM and SLS involve layer-by-layer consolidation of powder feedstock using a high-power laser to manufacture three-dimensional objects. With SLS, powder particles are partially melted by the laser to produce a porous, but coherent, structure; with SLM, the powder is completely melted to produce a dense part. For load-bearing craniofacial implants and patient-specific devices, both techniques offer high geometric freedom, strong mechanical properties, and the ability to fabricate complex lattice architectures [58,59]. The SLS/SLM process is particularly challenging when applied to bioceramics compared to metals or polymers. Many bioceramics cannot be directly melted in full (SLM). SLM can lead to cracking, phase changes, and loss of bioactivity in many bioceramics. Researchers have addressed this through hybrid or indirect methods: powder mixes containing polymeric binders or photopolymerized resins removed and sintered post-processing; laser-assisted slurry deposition with binder jets followed by thermal sintering; or the use of composite powders or glass-ceramic precursors that reduce the effective processing temperature. As a result of these approaches, porous, interconnected bioceramic scaffolds can be designed that manage shrinkage and maintain biofunctionality [32,58,60,61].

In addition to the advantages of SLS/SLM approaches for maxillofacial scaffolds, they also provide fine control over macroporosity, mechanical tailoring, and compatibility with digital preoperative workflows. It remains, however, difficult to achieve reliable micro- and nanoscale surface features that promote cell attachment, avoid thermal phase transformations that reduce bioactivity, control shrinkage during sintering, and ensure sterilizability and regulatory compliance. To achieve high bioactivity and a fine internal architecture, many groups favor extrusion-based or vat-photopolymerization methods for direct ceramic printing. At the same time, PBF routes are often explored to produce high-strength structural components and composite or metal–ceramic hybrid implants [6,62,63]. As a result, SLS and SLM produce patient-specific, mechanically robust constructs, but they require careful materials engineering and post-processing before they can be directly applied to pure bioceramics. For PBF designs to succeed in clinical trials, they must be combined with indirect ceramic processing or composite approaches that retain the biological advantages of ceramics while leveraging PBF’s structural advantages [58,64,65].

### 3.4. Binder Jet Printing

The binder jetting technique is an effective way of printing powder materials. Powder materials can be printed using binder jetting. Binder jetting relies heavily on powder particle size to determine powder flowability. For dry binder jetting, it is best to use large particles, as they are more flowable and have a lower surface area. In addition to affecting flowability, powder size also significantly impacts product quality. Many researchers have reported that the use of fine powder in binder jetting reduces surface roughness. A powder’s shape has less influence on its size than its shape. A spherical powder, however, has better flowability and lower friction than a faceted powder [66]. By selectively jetting liquid binder on successive thin layers of ceramic powder, Binder Jet Printing (BJP) builds a green part layer by layer. Powder spreading and binder deposition are separated in the technique, allowing printing at relatively high speeds and producing complex, patient-specific shapes without overheating the ceramic [58].

Dental and maxillofacial bioceramic scaffolds can benefit from BJP’s excellent geometric freedom, good dimensional control, and the ability to process a wide range of ceramic powders, provided appropriate powder packing and binder formulation are used. Because BJP parts are binder-filled powder compacts, post-processing is usually necessary, typically thermal debinding to remove organics, followed by high-temperature sintering to consolidate ceramic particles and restore mechanical strength. It is important to optimize these post-processing steps to balance scaffold strength and biological permeability, thereby achieving optimal porosity, microstructure, and mechanical performance [58]. Several limitations have been reported in the literature, including sensitivity to powder particle size distribution and packing density, residual porosity or weak interparticle bonds if sintering is not optimized, and the need to adjust binder chemistry to prevent contamination or excessive shrinkage when debinding. For craniofacial bone regeneration, BJP combined with careful thermal processing has demonstrated a versatile method for fabricating load-bearing ceramic components with controlled macro- and microporosity [67].

### 3.5. Hybrid and Multi-Material Printing Approaches

Dental and maxillofacial reconstruction requires scaffolds that are complex in terms of mechanics, structure, and biology. Hybrid and multi-material additive manufacturing can achieve this by combining two or more printing technologies or feedstocks. Ceramic prints can be combined with polymers or hydrogel components to create multifunctional architectures that cannot be achieved with single-material prints. By combining melt-extruded thermoplastics, photocurable resins, or cell-laden hydrogels with calcium phosphate or bioactive glass, load-bearing capability can be combined with a bioactive, cell-friendly microenvironment. By independently adjusting macro-porosity, micro-surface roughness, and local degradation rates, this multimodal strategy enables patient-specific grafts to balance immediate mechanical function and long-term biological integration [58]. It is also possible to integrate process-level workflows with hybrid workflows: using powder-based stereolithography, binder jetting, and material extrusion simultaneously or sequentially allows the printing of ceramic features alongside tougher polymer frames or sacrificial inks that form interconnected vascular channels when removed. In addition to improving printability for ceramics, these combinations expand the range of possible internal architectures and post-processing options. The advantages of these approaches include increased mechanical and biological resilience, the ability to incorporate growth factors or antibiotics at localized sites, and the ability to reproduce hierarchical bone structure more accurately in craniofacial models [58].

Ceramics and polymers have different thermal and chemical processing windows; ceramic sintering shrinks differently; adhesion between ceramic and polymer surfaces, sterilization, and regulatory complexity remain. Several factors need to be considered when addressing these issues, including selecting appropriate materials, adopting a design strategy, and implementing post-processing protocols that preserve bioactivity while ensuring dimensional stability. With further advancements in printing hardware, functional inks, and computational design for graded materials, hybrid bioceramic constructs for dental and maxillofacial reconstruction can become more clinically relevant and patient-specific [8].

### 3.6. Digital Workflow and Clinical Integration

In dental and maxillofacial practice, bioceramic scaffolds are both effective and durable when a digital workflow is implemented efficiently. To create accurate patient-specific implants (PSIs), prefabricated scaffolds, and surgical guides, a reproducible pipeline from image acquisition through design, manufacturing, and surgical execution is required [8]. Bone, dentition, and soft-tissue geometry can be collected using high-resolution volumetric imaging (CBCT/CT). The multimodal datasets are used to plan and design scaffolds based on anatomical information [68]. DICOM segmentation isolates the defect and adjacent structures, and segmentation and virtual surgical planning (VSP) enable defect reconstruction, osteotomy planning, and definition of implant boundaries. Prefabricating implants can be simulated using planning software. Porous architecture, implant shape, and fixation strategies can all be simulated before fabrication. Scaffold/PSI tools are designed using computer-aided design (CAD) for macrogeometry (patient fit and contours) and microarchitecture (pore size, interconnectivity, and gradient porosity). It is possible to design implants that incorporate channels for vascularization, fixation features, and surface texturing to enhance osseointegration [8,58]. A CAD model is converted into an STL or AMF file for printing. Based on the bioceramic printing method used, different slicing, support designs, and parameter settings will be applied. Printed scaffolds are usually debonded, sintered (ceramics), sterilized, dimensionally checked, and infiltrated or functionalized. Micro-CT, optical scanning, or mechanical testing are used to verify internal porosity. A digital plan is transferred to the OR using VSP outputs (surgical guides, templates) and patient-specific implants, enabling accurate osteotomies, scaffold placement, and fixation. Plan-execution loops are closed with intraoperative navigation and guides [8,58,68]. In complex cranio-maxillofacial reconstructions, VSP integration with 3D-printed bioceramic PSIs and porous scaffolds improves anatomical accuracy, reduces operating time and plate adaptation, and can improve aesthetic and functional predictability. Digitized workflows (scanning, designing, and printing in-house) result in shorter turnaround times and cost savings compared to outsourced manufacturing [69]. Designed and manufactured by external suppliers for complex ceramic processing that requires specialized furnaces. Imaging, segmentation, and preliminary CAD are performed in the clinical center; sintering and final finishing are performed in outsourced facilities. This model increases speed and iterative design, but requires investment in equipment, staff training, and quality systems to be successful; clinics with desktop printers for models and guides and access to ceramic processing are increasingly feasible [70].

Each bioceramic implant must comply with device regulations. Regional regulations remain heterogeneous, making multicenter adoption challenging. There are ongoing concerns about sintering shrinkage, brittle fracture risk, and mechanical stability early on in maxillofacial sites. Porosity gradients and fixation schemes should be carefully designed. It requires a multidisciplinary team of radiologists, biomedical engineers, and surgeons. Among the practical barriers are software and hardware incompatibilities. There are few large prospective cohorts or comparative trials, despite favorable case series; robust research is needed to predict long-term outcomes [8,58,68,70]. Machine-learning tools can automate segmentation, optimize scaffold architectures, and standardize workflows. Several early studies have shown that interoperator variability can be reduced and design time can be shortened. The integration of CBCT/intraoral scans, cloud-based CAD platforms, and surgical navigation software will reduce design-to-surgery intervals and improve reproducibility. A growing number of clinics are manufacturing scaffolds, guides, and models in-house for non-load-bearing or low-complexity reconstructions using ceramic printing and processing technologies [68,70,71]. Only through digital workflows can personalized bioceramic scaffolds be translated into clinics. As standardized protocols are adopted, regulatory clarity is achieved, clinical evidence is accumulated, and design is automated, these technologies will be utilized more widely in dentistry [8].

## 4. Types of Bioceramic Materials for 3D-Printed Dental and Maxillofacial Scaffolds

**Figure 2:**
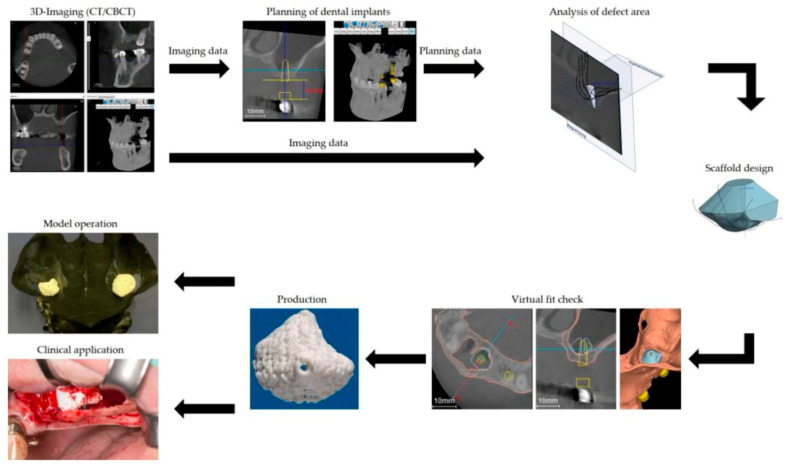
An overview of the workflow in a clockwise direction, starting at the top left. Three-dimensional radiographic data acquisition (**upper left**). Dental implant planning with yellow contours indicating the planned implant and its corresponding sleeve (**upper center**). A topographic and size analysis of the defect (**upper right**). The scaffold is designed virtually (**center right**). The yellow contour indicates the implant and the corresponding sleeve in the virtual check, while the light blue contour indicates the scaffold, and the brown contour indicates pristine bone in the transversal plane. The red arrows indicate the coronary plane (**bottom right**). Scaffolds are being manufactured (**lower center**). Modeling the defect situation in three dimensions (**left center**). The scaffold being applied to a patient (**lower left**) [74].

The intrinsic bioactivity and compositional similarity of bioceramic materials to native bone mineral make them crucial for bone tissue engineering. Choosing the right ceramic composition is crucial for successful clinical outcomes in dental and maxillofacial applications, where mechanical, aesthetic, and biological requirements are complex. Calcium phosphate, calcium silicate, and bioactive glass are among the main types of bioceramics used in 3D printing. A class’s physicochemical and biological characteristics determine its suitability for a given regenerative scenario. Biological scaffolds have been extensively used for bone tissue engineering because they are mechanically, structurally, and chemically similar to native bone apatite. Ceramic-based bone scaffolds are printed with customized shapes, pore sizes, and porosities, followed by high-temperature sintering. Size shrinkage may occur after sintering, but Young’s modulus and mechanical strength could increase drastically. Researchers created bioglass/Tx-CO scaffolds by 3D printing at room temperature with dextrin as the binder, followed by high-temperature sintering to achieve the desired shape and size [72]. According to Li et al. (2019), a novel bioceramic scaffold is being developed for drug loading and cell transport [73]. An extrusion 3D printer was used to create a hollow tube-shaped bioceramic rod. The sausage structure had an average diameter of 30 µm and was well-organized and uniformly layered. Due to its hollow tube structure, the scaffold facilitated blood vessel and bone formation [73]. In addition to adequate adaptation, Schulz et al. (2023) found that 3D-printed calcium phosphate cement scaffolds were stable, as evidenced by scaffold placement and postoperative maxillary bone rehabilitation following posterior implant surgery [74]. 3D printing, CBCT imaging, and CAD software were used to create the scaffolds. A scaffold was placed nine months after the dentition was reestablished with implants. It was observed that the implants had satisfactory primary stability during surgery, and there were no postoperative complications (Figure 2) [74].

To create a personalized geometry of bone defects, two different pore designs were applied: one rotated the layer by 60 degrees, and the other by 30 degrees. Scaffolds were manufactured via 3D-printed extrusion. As a result of implanting the scaffolds into artificial bone defects in the palates of mature Lewis rats, a robust structural support was demonstrated. The 60-degree angular orientation performed better than the 30-degree angular orientation. Although the scaffolds were precolonized with rMSCs, bone healing was not enhanced [75]. A study by Anderson et al. (2022) confirmed the clinical effectiveness of patient-specific grafts [76]. To evaluate the fit of printed scaffolds relative to their defect areas, CBCT and image-scanning models were superimposed using a best-matching algorithm. Despite the defect’s morphology or size, 3D-printed scaffolds showed an average fit deviation of just 0.27 mm after aligning the model of the defective area with the scaffolds [76].

### 4.1. Calcium Phosphate-Based Ceramics

#### 4.1.1. Hydroxyapatite Particle (HAp)

Biocompatible, osteoinductive, and osteoconductive, hydroxyapatite (HA) is widely used in 3D bioceramics. HA stimulates bone morphogenetic protein (BMP) and alkaline phosphatase production to induce osteogenesis. The drawbacks of HA include its brittle nature, limited load-bearing capacity, and slower degradation rate compared with other bioceramics. Composite scaffolds can be created by combining natural or synthetic polymers with HA to overcome these limitations [16]. Hydroxyapatite is the most biocompatible bioceramic containing inorganic components. Aside from being osteoconductive, HAps are low-resorbable, making them suitable for extensive bone grafting. HAp’s high brittleness makes it susceptible to fracture upon shock. Bioceramics are prone to fracture due to their inherent weakness and brittleness [77]. The proliferation and differentiation of preosteoblast cells on HDPE scaffolds treated with nitrogen plasma (N_2_) were studied by Park et al. in 2022 [77]. 3D printers were used to fabricate HDPE/n-HAp scaffolds. The particles were blended with HDPE and n-HAp at a 10% weight ratio. Nitrogen plasma reactive ion etching improved the biological response of preosteoblasts in vitro. After N_2_ plasma treatment, surfaces were characterized using Fourier transform infrared spectroscopy, scanning electron microscopy, and atomic force microscopy. We examined proliferating and differentiating preosteoblasts (MC3T3-E1) with ALP activity and MTT assays. Biochemical responses were improved in MC3T3-E1 cells treated with N_2_ plasma and n-HAp particles incorporated into the HDPE scaffolds [77].

Luo et al. (2018) synthesized titanium-substituted hydroxyapatite (Sr-HAP) nanoparticles using collagen type I and citrate as bi-templates [78]. They used 3D printing to create composite scaffolds with interconnected porous structures and nanoparticles that closely resemble natural bone minerals. Infrared, X-ray diffraction, scanning electron microscopy, and transmission electron microscopy measurements all showed a calcium-deficient HAP phase. Additionally, the Sr-HAP and HAP scaffolds were uniformly porous and structured. MC3T3-E1 cells cultured on scaffolds supplemented with strontium showed enhanced adhesion, proliferation, and alkaline phosphatase activity. A scaffold printed from composite materials was used to repair rabbit calvarial defects measuring 15 mm in diameter. Sr-HAP scaffolds demonstrated greater osteogenic potential after 12 weeks. As candidates for bone augmentation and regeneration, the Sr-HAP composite scaffolds are highly promising [78]. Kim et al. (2018) developed a 3D scaffold based on HAp to increase the osteoinductivity and compressive strength of bone [79]. 3D scaffolds were incorporated with an emulsion solution of polycaprolactone (PCL) and bone morphogenetic protein-2-loaded nanoparticles (BMP-2/NPs). Biocompatibility and osteogenic potential of the scaffold were evaluated using scanning electron microscopy and human mesenchymal stem cells. In vivo bone regeneration efficiency was assessed using a rabbit calvarial defect model. Three-dimensional HAp scaffolds containing NPs were created using PCL coating. To release BMP-2 gradually, uniformly distributed BMP-2/NPs were applied to the scaffold surface. A PCL coating was also applied to strengthen the scaffold. Cell proliferation, adhesion, and osteogenic differentiation properties increased as a result of PCL_BMP-2/NPs coating. As compared to uncoated scaffold-implanted scaffolds, PCL_BMP-2/NPs significantly enhanced the formation of new bone in vivo. In PCL and NPs emulsion solutions, BMP-2/NPs could be incorporated into the scaffold surface, thereby improving its compressive strength [79].

Bie et al. (2023) fabricated and investigated a 3D-printed artificial bone composite composed of HA-ZrO_2_-PVA [80]. These composites were composed of zirconia (ZrO_2_), hydroxyapatite (HA), and polyvinyl alcohol (PVA), with ZrO_2_ serving as a toughener and PVA solution as a binder. To obtain optimal 3D printing process parameters, the composites were optimized using a theoretical model of the extrusion process, followed by optimization of the spray head internal diameter, extrusion pressure, speed, and extrusion line width. Study results showed that biological bone scaffolds could be better fabricated using spray heads with a 0.2 mm diameter, extrusion pressures of 1–3 bar, and line spacing of 0.8–1.5 mm. The scaffolds met the biomechanical requirements for human bone, as determined by mechanical testing. According to scanning electron microscope observations, the scaffold sample exhibited a porosity of about 65%, which is conducive to chondrocyte growth and angiogenesis. In addition, chondrocytes proliferated by 100% more than their control counterparts after the composite materials were tested for biocompatibility. According to the results, the composite has good biocompatibility [80].

**Figure 3:**
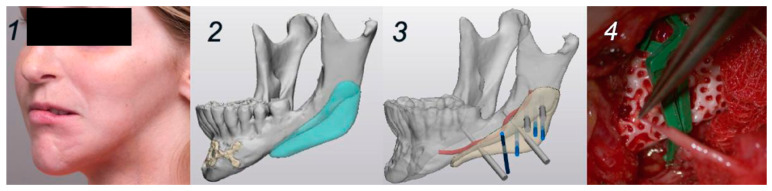
The planning and surgery of case 2. (1): Pre-surgery clinical situation, 45° left (2 and 3): PSI designs digitally. Designing the fixation screws in relation to the alveolar nerve digitally. (4): Placing and securing the PSI [69].

According to Verbist et al. (2024), these implants have both advantages and disadvantages regarding clinical outcomes [69]. The biocompatibility, biomechanical, and aesthetic properties of these implants were evaluated and compared with those of titanium and polyether-ether-ketone (PEEK) implants commonly used in orthopedic surgery. To illustrate the clinical outcomes of HA bioceramic PSI surgery, this article presents two clinical cases. A 19-year-old woman with Parry–Romberg syndrome had reconstructive surgery planned for the left mandibular angle as a result of left hemifacial asymmetry. In Mimics and Proplan CMFTM, the PSI and cutting guide for HA bioceramics were designed virtually. The screw holes were positioned to the alveolar nerve based on 3D CT images (Figure 3). There was no difference in the manufacturing process in either case. In the soft tissue reconstruction, peri-umbilical fat was lipofilled, and a flap of the Superficial Circumflex Iliac Artery Perforator (SCIAP) was used. Using a 3D-printed cutting guide, screw holes were drilled. As part of the prefixation process, the PSI was evaluated in its intended orientation. After the implant is inserted and fixed with two screws, the SCIAP flaps are harvested and anastomosed to the facial artery and vein (Figure 3). Fat harvested from the abdomen was lipofilled into the left upper lip. When integrated, they outperformed autologous bone grafts, PEEK, and titanium in terms of biomechanical properties. Our two clinical cases had satisfactory aesthetic results with good stability and no signs of infection or bone resorption. A six-month radiological follow-up showed osteogenic signs in both clinical cases. A HA bioceramic PSI mimics natural bone biomechanically and radiographically and has excellent biocompatibility. Compared with conventional biomaterials, they are well suited for restoring craniomaxillofacial defects with load-sharing [69].

#### 4.1.2. β-Tricalcium Phosphate (β-TCP)

Among the forms of tricalcium phosphate (TCP) are high- and low-temperature hydrophobic phases, which are used as bioceramics. This form is economical to prepare and stable at low temperatures. Due to its success in repairing postoperative defects in rabbits, β-TCP has received increasing attention. Its bioactivity is associated with calcium and phosphorus ions. These ions may form biological apatite deposits through partial dissolution and release. X-TCP demonstrated better biodegradability and resorption rates than HA, but it still could not achieve an equivalent rate of bone regeneration [16]. As demonstrated by Lee et al. (2020), 3D-printed heterogeneous, porous, wing-like scaffolds encouraged greater bone formation in dogs’ mandibles [81]. A variety of bone graft materials are available, so selecting the most appropriate technique for the specific reconstructed area is crucial [81]. In an experiment involving 3D printing, Entezari et al. (2019) developed bioceramic scaffolds with varying porosities and pore structures [82]. Pore sizes of 390 μm had superior bone formation in vivo, according to researchers. Additionally, the amount of new bone formed did not increase significantly when the scaffold pores exceeded 590 μm [82]. Researchers discovered that foamed scaffolds composed of spherical, concave macropores could be at least as effective as 3D-printed scaffolds in promoting bone regeneration and material resorption. This observation indicates that the macropore geometry significantly impacts biological responses and tissue regeneration. Spherical, concave macropores might provide a better microenvironment for cellular activity, facilitating efficient material resorption and enhancing bone regeneration [83,84].

Fahimipour et al. (2017) utilized a CAD model to fabricate a composite scaffold made of gelatin, alginate, and TCP in order to deliver VEGF to the vessel endothelium [85]. In standard Nordson cartridges, VEGF-loaded PLGA/alginate/TRCP paste was loaded into a water-based paste containing gelatin, alginate, and TRCP. The cartridge was then inserted into a scaffold replication system, and subsequent scaffold replication began. An optimized paste was developed and found to be suitable for printing as a bioink at room temperature, based on rheological characterization of various gelatin, alginate, and TCP formulations. The 3D-printed gelatin, alginate, and TCP scaffolds could be used to regenerate craniofacial defects by slowly releasing VEGF [85].

Using human dental pulp-derived mesenchymal stem cells (hDP-MSCs) for bone regeneration, Sajad Daneshi et al. (2024) constructed a 3D-printed nanohydroxyapatite/beta-tricalcium phosphate/collagen scaffold [86]. Angiogenesis and osteogenesis were examined in a rabbit mandibular defect model using this engineered construct. To demonstrate the critical and synergistic role collagen coating and incorporation of stem cells play in regeneration, three-dimensional scaffolds that were uncoated and loaded with stem cells, three-dimensional scaffolds that were collagen-coated, and scaffolds that were collagen-coated were included in addition to an empty defect. After euthanasia, X-rays, histological assessments, immunohistochemistry staining, histomorphometry, and reverse transcription polymerase chain reactions (RT-PCR) revealed substantial woven and lamellar bone in collagen-coated scaffolds used to print nHA/β-TCP scaffolds. When comparing the control group with the osteoblast, osteocyte, and osteoclast groups, histomorphometric analysis revealed significant increases in osteoblasts, osteocytes, and osteoclasts, as well as in bone area and vascularization. Fibroblasts and fibroocytes, however, were significantly increased in the control group. An RT-PCR analysis revealed significant increases in osteogenesis-related genes, including BMP2, ALPL, SOX9, Runx2, and SPP1. In critical-sized defect areas, 3D-printed scaffolds containing hDP-MSCs and collagen can promote bone regeneration [86].

### 4.2. Calcium Silicate-Based Ceramics

Despite its use as a biomaterial for only two decades, calcium silicate (Ca-Si) is well known as the main component of Portland cement in the construction industry. A monocalcium silicate (MCS) is composed of monocalcium oxide. In contrast, a dicalcium silicate (C2S) is composed of dicalcium oxide, whereas a tricalcium silicate (C3S) is composed of tricalcium oxide, and a pyrosilicate is composed of pyrosilicate. Three additional modifications are present in MCS. The high-temperature form, pseudowollastonite (pseudo-wollastonite), is rare in nature, while the two polymorphs, para-wollastonite and woolastonite, are more common [87]. A calcium-silicate-based material has been identified as a potential bioactive material for bone tissue regeneration due to its osseointegration properties. Ceramics, powders, and ceramic coatings can induce apatite formation upon implantation when in contact with biological fluids (SBF, saliva). Si^4+^ and Ca^2+^ are released from CS-based materials into the body, providing a wide range of health benefits. As a growth factor release promoter, calcium regulates osteogenesis, stimulates osteoclast differentiation, inhibits osteoporosis, and stimulates growth factor release. Additionally, Si^4+^ participates in the mineralization of early new bone formation and in the osteogenic differentiation of bone marrow mesenchymal stem cells. Calcium silicates have a complex structure that is influenced by several factors, including sintering temperature, precursors, composition, porosity, and fabrication method [88].

Cements based on tricalcium silicate are hydraulic bioactive materials widely used in dentistry and orthopedics as bone substitutes. The composition and manufacturing technique of various commercial TCS-based cements vary slightly. Two TCS-based dental cements with outstanding clinical performance are Biodentine and ProRoot White MTA (WMTA). Human dental pulp stem cells absorb Ca^2+^ from TCS-based biomaterials without killing them. As a consequence of stimulating human dental pulp stem cells with TCS-based cements, the increased Biodentine-linked Ca^2+^ load altered intracellular Ca^2+^ dynamics, which, in turn, led to changes in gene expression, cellular differentiation, and mineralization [87].

The bioceramic Akermanite (Ca_2_MgSi_2_O_7_) contains Ca, Mg, and Si and can have more controlled mechanical properties and degradation rates. The sol–gel method was used to synthesize pure, polycrystalline AKT particles with sizes of 5 to 40 μm. The ability of AKT to form apatite eventually led to its use as a scaffold for bone tissue engineering. BMSCs are stimulated to proliferate, adhere to each other, and differentiate when AKT is present. 3D-printed bioceramic scaffolds made from AKT powder also promote angiogenesis in addition to promoting angiogenesis [16]. The degradation of akermanite yields silicon (Si), calcium (Ca), and magnesium (Mg) ions as byproducts. Several studies have demonstrated the potential of Mg for peripheral nerve regeneration, in addition to the acknowledged bioactive effects of calcium, magnesium, and silicon ions in enhancing bone regeneration and mineralization [89].

The injectable bioactive composite microsphere developed by Gu et al. (2024) was designed to fit within the defect site and minimize injury [89]. Calcium calcitonin gene-related peptide (CGRP), a key hormone for bone repair, was released from sensory nerve cells when DPA microspheres were applied to bone marrow mesenchymal stem cells (BMSCs). In addition, the released CGRP modified epigenetic methylation to enhance osteogenic differentiation of BMSCs. By inhibiting EZH2 and increasing KDM6A, osteogenic genes such as Runx2 and Osx were activated, thereby reducing H3K27 trimethylation. A rat mandibular defect model demonstrates that peripheral nerve response facilitates bone regeneration through epigenetic modification by the bioactive microspheres. The study described a novel method for creating neuroactive osteoinductive biomaterials that could be used in subsequent clinical studies [89].

The ability of synthetic, 3D-printed Sr-HT-Gahnite scaffolds to repair large and load-bearing bone defects was examined in a study published by Li et al. (2019) [90]. As a comparison, sheep tibias were implanted with scaffolds at 3 and 12 months after implantation, and autografts served as controls. As a result of the scaffolds, substantial bone formation occurs, and defects are bridged after 12 months. A detailed examination of the bone–scaffold interface was performed using focused-ion-beam scanning electron microscopy and multiphoton microscopy. A fracture had developed in the newly formed bone, and the scaffold had degraded. An in silico analysis of scaffold strain energy distributions reveals that surgical fixation and mechanical loading influence long-term bone regeneration. 3D-printed Sr-HT-Gahnite scaffolds can also improve the repair of challenging bone defects and overcome the limitations of implantable bone grafts [90].

### 4.3. Bioactive Glass-Based Ceramics

The trade name of BG (bioactive glass) is 45S5 Bioglass^®^. BGs are mainly composed of Na_2_O, SiO_2_, CaO, and P_2_O_5_, and are mostly manufactured by melting and sol–gel processing. The fabrication of multifunctional scaffolds has also been made easier through 3D printing, and some functional agents can be directly incorporated into the bio-ink. There is significant potential for engineering bone tissue using BGs. As a result of the macroporous structure, nutrients and bone can be transported more easily. BMSCs are also proliferated and differentiated by their components (Ca, P, and Si). By releasing VEGF, BG can also create angiogenesis, making it more competitive [16]. According to Westhauser et al. (2016), different polymer-coated scaffolds made from 3D-45S5 bioglass were tested for their osteoinductive properties [91]. Implanting human mesenchymal stem cells (hMSC) into immunodeficient mice results in the formation of these scaffolds. The coatings used were gelatin, cross-linked gelatin, and poly (3-hydroxybutyrate-co-3-hydroxyvalerate). The new formation after eight weeks of implantation was evaluated using histomorphometry and micro-computed tomography. It should be noted, however, that bone regeneration was evident in every bioglass scaffold. Compared with other coated bioglasses, gelatin-coated scaffolds produced the most cells [91]. Recent studies by Nommeots-Nomm et al. (2018) reported bioglass scaffolds with pore sizes of 150 m and porosities of 41–43%, and measured compressive strengths of 32–48 MPa [92]. This scaffold is network-connected, similar to 45S5 bioglass. The NC in this process was closer to 45S5 by using a 50 mol% SiO_2_ composition. A bioglass scaffold made from 13 to 93 vol.% and a factory-produced scaffold were compared. Using two low-silica-content scaffolds, 3D porous scaffolds showed NC values similar to those of 45S5 bioglass. Pluronic F-127 binder can be used to bind bioactive glasses regardless of their composition or reactivity. A significant increase in bone regeneration speed was noted with scaffolds based on ICIE16 and PSrBG [92].

BG scaffolds can be constructed from marine natural sponges (MNS), but there is no in vivo data on their osteogenic properties. A BG-based scaffold obtained from MNS was evaluated for mechanical properties and seeded with human mesenchymal stem cells in two groups. Group A scaffolds were uncoated (Group A), and Group B scaffolds were coated with gelatin (Group B). The scaffolds were histomorphometrically analyzed before implantation and after explantation using micro-computed tomography. Both scaffolds exhibited bone formation. During the implantation period, scaffold volume increased significantly in Group B scaffolds (8.95%, *p* = 0.039), while scaffold volume increased nonsignificantly in Group A scaffolds (5.26%). With gelatin coating, compression strength increased significantly (*p* = 0.029). An osteogenic response can be greatly enhanced in vivo by coating BG-based scaffolds with gelatin [93].

## 5. Personalization in Scaffold Design

A contemporary tissue engineering approach emphasizes patient-specificity, especially in dental and maxillofacial reconstruction, given the complexity of anatomy, functional requirements, and patient variability. 3D printing, advanced medical imaging, and bioceramic materials enable the design and fabrication of scaffolds tailored to patients’ anatomy, biology, and mechanical requirements. Compared with conventional “one-size-fits-all” grafting approaches, customized scaffolds can improve clinical outcomes, reduce complications, and enhance functional integration. During chewing, speaking, and expressing facial expressions, the craniofacial region faces unique challenges due to its intricate geometry and aesthetic significance. There is a wide range of irregular shapes caused by trauma, tumor resections, congenital anomalies, and periodontal disease. Traditional autografts and alloplastics require extensive intraoperative modifications, compromising structural integrity. By using patient-specific imaging data to fabricate bioceramic scaffolds, these limitations can be addressed [94]. Similarly, millimeter-level accuracy is crucial for implant stability and long-term success in dental applications such as alveolar ridge augmentation, periodontal regeneration, and maxillary sinus floor elevation. Optimizing scaffold geometry, porosity, and degradation kinetics according to individual patient needs can enhance osteointegration and reduce implant failure rates [95,96].

Personalization of bioceramic scaffolds relies heavily on digital workflows that integrate medical imaging, CAD, and additive manufacturing. Dental and maxillofacial defects can be captured using high-resolution imaging modalities such as CBCT and C. Using segmentation software, the datasets are converted into digital 3D models, which enable accurate reconstruction of each patient’s defect morphology [97].

As scaffold architectures are refined using CAD tools, defect contours can be customized, and pore networks can be designed to support vascularization. Advanced software platforms reduce the risk of design failure by simulating mechanical performance, stress distribution, and degradation behavior. The digital-to-physical pipeline significantly reduces design-to-implant time and improves reproducibility, making personalized scaffolds more feasible for clinicians to use [98]. Scaffold design personalization includes anatomical customization, which is clinically relevant and immediate. Scaffolds with high geometrical fidelity, including mandibular contours, alveolar ridges, and orbital walls, can be fabricated using 3D printing techniques such as SLA, DLP, and robocasting. In this way, micromotion is minimized, and stabilization is promoted by ensuring intimate contact between the scaffold and host bone [8,99]. Scaffolds used in dental implantology can accommodate implant fixtures and support bone regeneration around implants. A patient-specific ridge augmentation scaffold provides superior volumetric stability for patients with vertically deficient ridges. In addition, these designs can reduce postoperative morbidity and increase patient comfort by allowing for minimally invasive surgery [95,100].

Personalization extends beyond external geometry to the internal microstructure of bioceramic scaffolds, which regulates biological responses. Pore size, shape, interconnectivity, and gradient distribution can be customized for different defect sizes, anatomical locations, and healing capacities. Macropores facilitate bone ingrowth and vascularization, while protein adsorption and osteogenic cell attachment are facilitated by micropores [6,8,18,86,101]. Functionally graded porosity is incorporated into advanced design strategies to mimic the hierarchical structure of native bone. The denser regions of a structure can withstand mechanical loads, while the porous zones can facilitate rapid tissue infiltration. The use of gradient scaffolds in mandibular and maxillary reconstructions is particularly beneficial, since both load-bearing and regenerative functions are required. The porosity design can also be tailored to the patient’s age, bone quality, and other systemic conditions that may affect healing [102]. Personalization can be further enhanced by selecting the right material and composition. Calcium phosphates, bioactive glasses, and calcium silicate-based ceramics have tunable properties that can be tailored to clinical requirements. It may be necessary to adjust ceramic composition to control degradation rates, ion release profiles, and mechanical strength when designing a customized scaffold [1,66,88,103]. Angiogenesis and osteoblast differentiation can be stimulated by bioactive glass scaffolds that release osteogenic ions such as silicon, calcium, and phosphate. Similarly to calcium silicate-based ceramics, calcium silicate ceramics can enhance early-stage mineralization and antibacterial properties, making them suitable for infected or high-risk sites. The personalization spectrum is further enhanced by composite scaffolds that incorporate polymers and doped ceramics to balance brittleness, elasticity, and bioactivity [1].

Biologically personalized scaffolds may be used in dental and maxillofacial applications to address site-specific healing challenges, such as limited vascularity in large mandibular defects or chronic inflammation in periodontitis. Using scaffold surface modification and controlled-release systems, local immune responses can be modulated, and favorable healing environments can be promoted. Regenerative dentistry is moving toward precision medicine through these approaches [104,105]. Bioceramic scaffolds are closely linked to personalized surgical planning for clinical implementation. To simulate scaffold placement and fixation strategies, as well as implant integration, surgeons use digital planning tools. The use of patient-specific surgical guides and fixation plates enhances accuracy and reduces intraoperative variability. The use of customized 3D-printed bioceramic scaffolds for alveolar ridge augmentation and maxillofacial defect reconstruction has demonstrated improved fit, reduced operative time, and satisfactory bone regeneration in several clinical studies. Clinical adoption, however, is limited by regulatory challenges, cost considerations, and the lack of standardized manufacturing protocols. Personalized bioceramic scaffolds are likely to become a vital component of regenerative dental and maxillofacial therapies as these technologies mature [6,8,89,95].

## 6. Clinical and Preclinical Applications

With 3D printing, scaffold geometry, internal architecture, and material composition can be precisely controlled, enabling the fabrication of patient-specific bone graft substitutes. Bioceramics are among the most extensively studied materials for bone regeneration due to their biocompatibility, osteoconductivity, and customized degradation profiles. In dental and maxillofacial bone regeneration, implantology, and the reconstruction of critical bone defects, these features make them ideal for clinical and preclinical applications. In recent years, personalized bioceramic scaffolds have advanced beyond conventional bone grafts to accelerate healing and minimize donor site morbidity and surgical complications [106,107,108].

After tooth extraction or trauma, the alveolar bone must be restored to achieve dental implant success. For aesthetic zones where bone volume is critical to functional and cosmetic outcomes, insufficient alveolar ridge volume presents a significant challenge in implant dentistry. In addition to their limited availability, immunogenic responses, and unpredictable resorption rates, traditional grafting materials (autografts, allografts, and xenografts) have other limitations. The goal of tissue engineering strategies that incorporate biodegradable 3D-printed scaffolds is to overcome these limitations by providing customized bone regeneration templates that integrate with the host tissue and encourage new bone formation [109].

A key clinical objective of scaffold-guided bone regeneration (SGBR) is to support cellular infiltration, vascularization, and osteogenesis using a customized scaffold that fits precisely to the defect morphology. Several studies have demonstrated that polycaprolactone (PCL) scaffolds, specifically printed for patients and combined with bioactive ceramics such as tricalcium phosphate, can effectively promote alveolar bone formation. This study describes the use of a 3D-printed PCL resorbable scaffold to augment alveolar bone and place dental implants in a 46-year-old patient. Personalized scaffolds have been shown to support clinical bone regeneration in humans, as indicated by a volumetric bone gain of 364 mm3, histological evidence of new bone formation throughout the defect, and a primary stability of the dental implant [110,111,112].

Bioceramic composition and scaffold architecture are strongly associated with biological outcomes, according to preclinical studies. In vivo, bioceramic scaffolds with interconnected channel networks produced using extrusion-based 3D printing have been shown to enhance bone regeneration, presumably due to improved nutrient transport and cell infiltration [106]. According to these findings, regenerative capacity can be maximized by extending personalization beyond overall shape and incorporating microarchitecture engineering within. For durable alveolar ridge regeneration prior to implant placement, porous materials can mimic trabecular bone and facilitate osteogenesis and angiogenesis [106].

Trauma, resection of tumors, congenital anomalies, or infection can cause bone defects in the maxillofacial region. In a nutshell, autologous bone grafting remains the gold standard, but it has several limitations, including donor site morbidity, prolonged operative times, and variable resorption rates. In addition to providing biocompatible and osteoconductive materials, customized bioceramic scaffolds offer a promising alternative for reconstructing complex defect geometries. According to a scoping review of clinical studies up to 2024, 3D-printed bioresorbable scaffolds designed using virtual surgical planning have been successfully used to repair a variety of maxillofacial bone defects, including those of the mandible, maxilla, zygomatic bone, and frontal bone. A successful bone union and dental implant placement were achieved without the need for additional guided bone regeneration procedures using these scaffolds, which demonstrated favorable dimensional compatibility, structural support, and bone formation-promoting properties. Additionally, most of these scaffolds demonstrated biocompatibility and few serious adverse events, making them attractive options for clinical reconstruction [113].

The clinical success of several cases and series has been documented. In some cases, composite scaffolds of alkaline phosphates and calcium phosphates are used to reconstruct defects of varying complexity. After 6–24 months, the scaffolds develop partial or complete union with the host bone, depending on the degree of deformity. Infection and wound dehiscence have been reported as postoperative complications, highlighting the importance of careful surgical planning and follow-up [113].

Clinical successes have been documented in several case reports and series. Digital workflows improve surgical predictability and aesthetic outcomes by ensuring scaffold fit and minimizing intraoperative adjustments. Over a period of 6–24 months, partial or full bone union has been demonstrated with defects of varying complexity. Wound dehiscence and infection have been reported following surgery, emphasizing the need for meticulous surgical planning [114]. Additionally, digital planning and 3D printing enable the fabrication of surgical guides, facilitating precise osteotomies and implant placement, which are crucial to functional rehabilitation and aesthetics [114].

For dental implants to be successful, there must be sufficient and healthy bone at the implant site. Increasingly, 3D-printed bioceramic scaffolds are being explored to preserve sockets, enhance ridge morphology, and facilitate guided bone regeneration (GBR) for implants. Rapid ridge resorption following tooth extraction may compromise the placement of future implants [106,109,110,114]. Despite limited high-quality randomized clinical trials, preliminary studies suggest that personalized scaffolds may improve ridge preservation and bone augmentation outcomes compared with traditional grafting techniques. As a result of synergistic use of scaffolds with guided bone regeneration membranes or growth factors, these benefits include better dimensional stability, reduced vertical bone loss, and favorable implant stability. To establish definitive clinical guidelines on optimal scaffold materials, porosity profiles, and combinations with biological agents for routine implantology, further rigorous trials and systematic reviews are required [114].

To enhance bone regeneration kinetics and functional outcomes, translational research in animal models continues to refine scaffold designs and material compositions. The osteogenic response and in vivo vascularization can be significantly enhanced with advanced bioceramic constructs compared to traditional ones, according to studies. As a result of these designs, nutrient transport and cellular migration are improved by precisely engineered channel networks that mimic native bone architecture [66]. Besides osteoconductivity, recent preclinical work suggests 3D-printed bioceramics can modulate the local immune microenvironment, which is crucial to bone healing, particularly in aging or compromised individuals. In the early stages of bone healing, tailored bioceramic scaffolds may influence macrophage polarization and inflammatory pathways [115]. To further stimulate osteogenesis and angiogenesis, bioactive molecules, such as growth factors, peptides, or pharmacological agents, can be incorporated into 3D-printed bioceramic constructs. Multifunctional designs aim to accelerate bone regeneration, both structurally and biologically [58]. While bioceramic scaffolds have shown promise in clinical and preclinical studies, several challenges remain: their osseointegration is often slower than that of native bone, and tissue responses are variable. To enhance biological performance, surfaces can be functionalized, bioactive agents incorporated, and porosity gradients optimized to mimic bone microenvironments [58]. For scaffolds to function as effectively as native bone, their mechanical properties and degradation rates must be matched to those of native bone. Degradation that occurs too slowly may hamper natural remodeling, while degradation that occurs too rapidly may compromise structural stability. Blends of materials are being developed to balance these characteristics [18]. Personalized bioceramic scaffolds must be validated through large-scale randomized clinical trials compared to gold-standard treatments, while case reports and small series provide valuable insights. As clinical evidence becomes more robust, optimal indications, scaffold designs, and long-term results will become clearer [114].

## 7. Future Directions

Despite significant progress in three-dimensional printing and personalized bioceramic scaffolds for dental and maxillofacial applications, several critical research and translational challenges remain, offering clear directions for future research. Additively manufactured bioceramic constructs will achieve their full clinical potential only if these areas are addressed. The development of next-generation bioceramic composites that combine enhanced mechanical strength with controlled biodegradation and bioactivity should be the focus of future research. Several strategies may improve fracture resistance and toughness, especially in load-bearing craniofacial implants, including multiphase ceramics, nanoparticle reinforcement, and polymer–ceramic hybrids. Additionally, surface functionalization with bioactive molecules, antimicrobial agents, or osteoinductive ions can accelerate bone regeneration and reduce infection risk. A major limitation of current bioceramic scaffolds is their insufficient vascularization after implantation. Scaffold designs with gradient porosity, interconnected microchannels, and hierarchical pores should be explored in future studies to promote angiogenesis. It may be possible to enhance vascular ingrowth and long-term tissue integration further by incorporating angiogenic growth factors, extracellular matrix-inspired coatings, or co-printing with endothelial-supportive biomaterials. Using multi-material and 4D printing technologies to create biomimetic scaffolds with spatially controlled mechanical and biological properties is a promising direction. It would be possible to better replicate the heterogeneous structure of craniofacial tissues by combining bioceramics with biodegradable polymers or hydrogels in a single construct. It may also be possible to improve surgical handling and post-implantation adaptation using 4D-printed scaffolds that can transform shape or control degradation in response to physiological stimuli. As digital imaging, computer-aided design, and artificial intelligence (AI)-assisted modeling are integrated, personalization and clinical outcomes will be enhanced. By analyzing patient-specific anatomical and biomechanical data, AI could optimize scaffold geometry, porosity, and mechanical performance. Regulatory approval and clinical scalability can also be enhanced through standardized digital workflows. To validate 3D-printed bioceramic scaffolds as safe, effective, and cost-effective, robust, long-term in vivo studies are needed. Studies comparing conventional grafting materials to standardized testing protocols and clinically relevant animal models should be prioritized in the future. Streamlining translation from bench to bedside will require collaboration among material scientists, clinicians, and regulatory bodies. In addition, future research should focus on how to manufacture bioceramic scaffolds at a scalable and sustainable level. Biomedical manufacturing may also become increasingly dependent on environmentally friendly raw materials and energy-saving printing processes.

## 8. Limitations

Despite the rapid progress and promising outcomes reported in the field of three-dimensional (3D) printing of personalized bioceramic scaffolds for dental and maxillofacial applications, several limitations should be considered when interpreting these results. The first disadvantage of this article is that it is a narrative rather than a systematic review, so no predetermined protocol for literature search, study selection, or data extraction has been followed. Thus, some relevant studies—particularly unpublished data or negative findings—might have been excluded due to selection bias. Even though recent and influential publications were incorporated, the conclusions drawn should be viewed as descriptive rather than quantitative. There are relatively few well-designed clinical trials evaluating 3D-printed bioceramic scaffolds for dental and maxillofacial reconstruction, a limitation of the current literature. It remains unclear whether these scaffolds in humans are biologically effective, degradation-resistant, or safe over the long term. Experiments with preclinical models and human patients are different in terms of defect size, anatomical complexity, loading conditions, and healing environments, limiting their direct translation into clinical practice. Thirdly, the composition and printing techniques of materials, as well as the protocols for post-processing, differ considerably between studies. It is challenging to compare results directly due to variations in bioceramic formulations, printing parameters, sintering conditions, and scaffold architectures. A lack of standard fabrication and characterization protocols limits reproducibility and complicates the development of consensus regarding optimal scaffold designs. Printed bioceramic scaffolds also face limitations in mechanical performance and functional validation. Often, studies focus primarily on porosity, compressive strength, and bioactivity under static lab conditions, which may not adequately reflect oral and maxillofacial biomechanical environments. Underreported factors such as fatigue resistance, long-term mechanical stability, and performance under cyclic loading are crucial to clinical success, particularly in load-bearing applications. In addition, the literature on vascularization, soft tissue integration, and the immune response does not fully address these challenges. Advanced scaffold designs and bioactive modifications can enhance angiogenesis and osteointegration, but robust in vivo evidence of vascularized bone regeneration remains lacking. This gap hampers the development of large or complex patient-specific scaffolds. Cost, regulatory approval, and manufacturing scalability are also barriers to widespread adoption from a clinical and translational perspective. Patient-specific 3D-printed implants are challenging to implement routinely in clinical settings because of high equipment costs, technical expertise requirements, and regulatory uncertainty. Further, there is a lack of long-term follow-up data regarding clinical outcomes, complication rates, and cost-effectiveness. Additionally, some emerging methods, hybrid materials, and biofabrication strategies are not yet fully represented in the available literature due to the rapid evolution of 3D printing technology. As a result, the review reflects the current state of knowledge but may not capture the full potential of ongoing innovations in the fabrication of personalized bioceramic scaffolds. Taking these limitations into account, this narrative review should be viewed as an overview rather than definitive clinical advice. For 3D-printed bioceramic scaffolds to be safely and effectively translated into dental and maxillofacial applications, standardized methodologies, long-term clinical trials, and interdisciplinary collaboration should be prioritized in future research.

## Data Availability

The original contributions presented in this study are included in the article. Further inquiries can be directed to the corresponding authors.
